# Amelioration of inflammatory bowel disease by *Bifidobacterium animalis* subsp. *lactis* XLTG11 in combination with mesalazine

**DOI:** 10.3389/fmicb.2024.1472776

**Published:** 2024-12-04

**Authors:** Weiwei Ma, Yanan Wu, Xinyue Lin, Liping Yang, Lili Huang

**Affiliations:** College of Pharmacy, Heilongjiang University of Chinese Medicine, Harbin, China

**Keywords:** *Bifidobacterium animalis* subsp. *lacti*, mesalazine, IBD, intestinal microorganisms, SCAFs

## Abstract

The treatment of inflammatory bowel disease (IBD) remains challenging and significantly impacts both patients and their families. This study evaluated the role of *Bifidobacterium animalis* subsp. *lacti* XLTG11 (XLTG11) in combination with mesalazine (5-ASA) in the improvement of IBD. The results demonstrated that the XLTG11+5-ASA group exhibited superior recovery compared to both the XLTG11-only group and the 5-ASA-only group. The XLTG11+5-ASA group significantly reduced myeloperoxidase activity (MPO), attenuated colonic tissue damage, lowered the levels of lipopolysaccharides (LPS) and D-lactic acid (D-LA), and decreased intestinal permeability. Furthermore, it upregulated the mRNA expression of Claudin-1, Occludin, ZO-1, and MUC2, which contributed to the protective effect on intestinal barrier function. Additionally, the XLTG11+5-ASA group significantly increased the levels of anti-inflammatory cytokines while decreasing pro-inflammatory cytokine levels. Notably, treatment with the XLTG11+5-ASA group significantly increased levels of acetic, propionic, and butyric acids, as well as the relative abundance of beneficial bacteria such as *Bifidobacterium* and *Lactobacillus*, while decreasing the relative abundance of *Enterococcus*, *Enterobacteriaceae*, and *Clostridium perfringens*. The results indicate that the combination of XLTG11 and 5-ASA was more effective in treating IBD than either treatment alone, significantly improving IBD-related symptoms and providing a scientific basis for future clinical applications.

## 1 Introduction

Inflammatory bowel disease (IBD) is a chronic, recurrent condition that severely compromises the barrier function of the intestinal mucosa. The two main forms of IBD, Crohn’s disease (CD) and ulcerative colitis (UC), are characterized by prolonged, continuous treatment requirements (Gajendran et al., 2018). The precise pathogenesis of IBD remains unclear, but the disease is known to be influenced by a variety of factors, including genetic susceptibility, mucosal barrier damage, intestinal microecological imbalances, and dysregulated immune system responses (Seril et al., 2003). IBD often manifests at a young age, and due to its persistent and often relapsing nature, patients typically require long-term treatment, which must be adjusted based on the individual’s condition and the complications arising from previous medications. This not only places a significant economic burden on patients and their families but also on society at large.

IBD is characterized by low mortality but high morbidity, recurrence rates, and disability, making it a heavy burden on public health systems as well as healthcare resources. These factors highlight the urgent need for effective therapeutic strategies to alleviate the suffering of IBD patients during both active and remission phases (Li et al., 2023). IBD has become one of the most challenging diseases worldwide (Loftus, 2004). While IBD was once thought to be primarily a disease of highly developed countries, the incidence of new cases has been rising globally. As of 2020, it is estimated that three million people in Europe, three million in the United States, and over 80,000 in Australia are affected by IBD (Jakubczyk et al., 2020). In recent years, the global incidence of IBD has continued to rise (Molina-Infante et al., 2018), posing a growing challenge to public health and having a profound impact on healthcare systems around the world.

Dextran sodium sulfate (DSS) is a sulfated polysaccharide derived from dextran and sulfated glucose. It is commonly used to induce colitis in experimental models due to its wide applicability and low cost. The DSS-induced colitis model is one of the most frequently employed methods for studying intestinal inflammation. DSS induces enteritis by disrupting the structural integrity of intestinal epithelial cells, increasing intestinal permeability, and allowing biomolecules, including DSS itself, to penetrate the intestinal lining. In DSS-induced colitis, the intestinal mucosal epithelium loses its mucin content, leading to programmed cell death (necrosis) of epithelial cells and triggering an inflammatory response that mirrors the pathology seen in ulcerative colitis (Alkahtani et al., 2013; Goyal et al., 2014).

Traditional treatments for inflammatory bowel disease (IBD) include mesalazine and immunosuppressants. Mesalazine, also known as aminosalicylic acid (5-ASA), is a highly effective, safe, and well-tolerated drug, particularly for treating mild to moderate ulcerative colitis. By delivering high doses of active compounds directly to the site of inflammation through oral or rectal administration, mesalazine allows the drug to be absorbed in the distal colon, minimizing the risk of systemic adverse effects. Therefore, 5-ASA is often used as a first-line treatment for colitis. However, long-term use of these drugs may lead to complications, and many IBD patients on 5-ASA therapy may need to discontinue the medication. Side effects of 5-ASA include nausea, vomiting, headache, abdominal pain, rash, and, in some cases, exacerbation of colonic inflammation (Tsujii et al., 2022). Additionally, mesalazine-induced nephritis and renal dysfunction have been reported, independent of dosage or formulation. Consequently, it is recommended that renal function be regularly monitored in IBD patients on continuous oral 5-ASA maintenance therapy (Williams et al., 2011).

Probiotics are biologically active microorganisms that confer health benefits to the host when consumed in adequate amounts (Juarez et al., 2013; Evivie et al., 2017). Studies have shown that probiotics generally cause fewer adverse reactions and do not interfere with nutrient absorption (Boussenna et al., 2015; Sun et al., 2016). Furthermore, certain probiotics are capable of secreting anti-inflammatory cytokines, making them a promising therapeutic option for chronic inflammatory diseases, including inflammatory bowel disease (IBD) (Wu et al., 2022). Among the most commonly used probiotics are bacteria such as *Lactobacillus acidophilus* and *Bifidobacterium bifidum*.

Previous studies have demonstrated that *Bifidobacterium animalis* subsp. *lactis* XLTG11 has both preventive and therapeutic effects on colitis (Wang et al., 2021). Therefore, the aim of this study was to investigate the potential of *Bifidobacterium animalis* subsp. *lactis* XLTG11 in combination with mesalazine to ameliorate inflammatory bowel disease (IBD). Specifically, the study aimed to evaluate the effect of this combination therapy in alleviating IBD through measurements of changes in the intestinal microbiota, histopathological analyses, intestinal barrier function, cytokine profiles, and myeloperoxidase activity.

## 2 Materials and methods

### 2.1 Animal grouping and feeding

Sixty BALB/c mice (Beijing Viton Lihua Laboratory Animal Technology Co., Beijing, China, License No: SCXK (Beijing) 2016–0006) were housed in an animal facility at 22°C with 10–60% humidity and a 12-h light/dark cycle. The mice were acclimatized for 1 week, during which they were fed a standard diet and provided free access to water. Following acclimatization, the mice were randomly assigned to five groups of 12 animals each: the normal control (NC) group, model control (MC) group, XLTG11 group, 5-ASA group, and XLTG11+5-ASA group.

During the experimental phase, the NC group was maintained on a normal diet and water, with daily gavage of sterile PBS (0.2 mL). The MC, XLTG11, 5-ASA, and XLTG11+5-ASA groups received a 3% DSS aqueous solution for 7 days to induce an ulcerative colitis model, followed by daily gavage of sterile PBS (0.2 mL) in the MC group. The XLTG11 group received daily gavage of *Bifidobacterium animalis* subsp. *lactis* XLTG11 (5 × 10^8^ CFU/mL, 0.2 mL, provided by the Food Microbiology Laboratory, Northeast Agricultural University); the 5-ASA group received daily gavage of 5-ASA (0.4 g/kg/day, Sigma-Aldrich, Shanghai, China, 0.2 mL); and the XLTG11+5-ASA group received both daily gavage of XLTG11 (5 × 10^8^ CFU/mL, 0.2 mL) and 5-ASA (0.4 g/kg/day, 0.2 mL) for 2 weeks.

Ethical approval for this study was granted by the Animal Ethics Committee of Northeast Agricultural University (protocol number: NEAUEC20230435). All animal procedures were performed in accordance with the guidelines for good animal practice (Percie du Sert et al., 2020).

### 2.2 Changes in body mass

Fasting body mass was measured at the beginning of the experiment, at the end of the experiment, and every 7 days during the intervention period.

### 2.3 Measurement of colon related indicators

After the mice were sacrificed, their colon tissues were collected. The levels of cytokines (TNF-*α*, IL-1β, IL-6, and IL-10) in the colon tissues were determined using the corresponding ELISA kits (Jianjian Biological Co., Nanjing, China), following the manufacturer’s instructions.

The MPO (Myeloperoxidase) activity measurement procedure is as follows: Mouse colon tissue was weighed accurately and added to homogenization medium at a tissue-to-medium ratio of 1:19 to prepare a 5% tissue homogenate. A 0.9 mL aliquot of the 5% homogenate was then taken, and each reagent was added according to the instructions provided in the MPO biochemical kit. Finally, the absorbance was measured at 460 nm. The MPO activity was calculated using the following formula:


$$\begin{array}{l} \mathrm{MPO}\;\mathrm{Activity}=\left(\mathrm{Measured}\;\mathrm{OD}-\mathrm{Control}\;\mathrm{OD}\right)\\ \;\;\;\;\;\;\;\;\;\;\;\;\;\;\;\;\;\;\;\;\;\;\;\;\;\;\;\;\;\;\;\;\;\;\;\;\;\;\;\;/\mathrm{Sample weight}\;\left(\mathrm{grams}\right). \end{array}$$

In addition, mouse distal colon tissue was fixed in a 4% (v/v) formaldehyde solution, embedded in paraffin, deparaffinized with xylene, and stained with hematoxylin–eosin (HE). Histopathological changes in the colon were observed under a light microscope.

### 2.4 Detection and measurement of intestinal permeability

Hematoxylin–Eosin (HE) staining was performed to observe the pathological structure of the intestine, using commercial kits (Herpine Biotechnology Co., Shanghai, China). Levels of LPS (lipopolysaccharide) and D-lactic acid in the intestine were quantified using specific kits (Konodi Biotechnology Co., Quanzhou, China).

### 2.5 Measurement of the intestinal barrier

The intestinal barrier was assessed by measuring the mRNA expression levels of genes related to intestinal mucins (MUC2) and tight junction proteins (ZO-1, Occludin, and Claudin-1) using quantitative reverse transcription polymerase chain reaction (qRT-PCR) with commercial kits (Promega Biotechnology Co., Beijing, China). The primer sequences used for qRT-PCR are listed in Table 1.

**Table 1:** Primer sequences used for quantitative polymerase chain reaction.

**Genetics**	**Forward primer**	**Reverse primer**
ZO-1	GCGAACAGAAGGAGCGAGAAGAG	GCTTTGCGGGCTGACTGGAG
Occludin	TGGCTATGGAGGCGGCTATGG	AAGGAAGCGATGAAGCAGAAGGC
Claudin1	GCTGGGTTTCATCCTGGCTTCTC	CCTGAGCGGTCACGATGTTGTC
MUC2	TGCTGACGAGTGGTTGGTGAATG	TGATGAGGTGGCAGACAGGAGAC

### 2.6 Determination of short-chain fatty acids

Accurately weigh 0.80 ± 0.010 g of cecal content and place it in a fecal sample container. Process the sample using the HALO-F100 fecal processor to prepare a 10% suspension. Take 500 μL of the suspension (or 500 μL of liquid culture medium if applicable) and transfer it to 1.5 mL centrifuge tubes. Add 100 μL of croscarmellose sodium metaphosphoric acid solution to each tube, then freeze the samples at −30°C for 24 h.

After thawing, centrifuge the samples at 8,000*g* for 3 min at 4°C to remove proteins and other impurities. Carefully collect the supernatant, and filter it through a 0.22 μm aqueous filter. The filtered supernatant is then ready for measurement using the appropriate instrument.

### 2.7 Determination of intestinal microorganisms

The feces of each group of mice were taken, and total DNA was extracted from the samples According to the commercial kit steps (Tengen Biochemical Technology Co., Beijing, China), and the V3-V4 regions of 16S rDNA were amplified using the bacterial genome as a template using high-fidelity enzyme with the primers V3F: 5’-CCTACGGGGNGGCWGCAG-3’ V4R: 5’-GACTACHVGGGT ATCTAATCC-3′, and the tag sequence was added at the 5′-end of the primer. V4R: 5’-CCTACGGGGNGGCWGCAG-3’ V4R: 5’-GACTAC HVGGGTATCTAATCC-3′, and tag sequence was added at the 5′ end of the primer. PCR amplification was performed, the products were purified, quantified by Qubit 3.0 Fluorescence Photometer, Miseq library was constructed and sequenced using Illumina Miseq platform. Filtered data references were spliced and sequenced sequences without overlapping relationships were removed using FLASH (V1.2.7) software, and chimeric sequences were removed using the UCHIME algorithm to obtain high-quality sequence tags. Sequence tags with 97% sequence homology were clustered using Uparse software to obtain representative sequences of the operational taxonomic unit (OTU). The sequences were compared with the GreenGene database using PyNAST software, and the OTUs were annotated with taxonomic information. The phylogenetic tree was generated using MUSCLE software and the abundance information of OTUs was normalized. Species composition was analyzed by QIIME software (V1.7.0) and R software (V3.4.1).

### 2.8 Statistics and analysis of data

SPSS statistical software was used to analyze the experimental data. The experimental data were statistically analyzed using SPSS 17.0 software, applying one-way ANOVA Duncan’ test for comparison between multiple groups of data, with *p* < 0.05 as significant, *p* < 0.01 as highly significant, and *p* > 0.05 as non-significant. Graphs were plotted using GraphPad Prism 9.5.

## 3 Results

### 3.1 Changes in body weight and MPO viability in mice

To assess the effects of *Bifidobacterium animalis* subsp. *lactis* XLTG11 and 5-ASA on body weight, the initial and final body weights of the mice were recorded. The results are shown in Table 2. It was observed that the final body weight of mice in the MC group was significantly reduced compared to the NC group (*p* < 0.01). In contrast, all treatment groups (XLTG11 group, 5-ASA group, and XLTG11+5-ASA group) showed a significant increase in final body weight compared to the MC group. Among these, the combination treatment of XLTG11+5-ASA resulted in a significantly greater increase in final body weight (*p* < 0.01), while the individual treatments with either XLTG11 or 5-ASA also led to a significant increase in body weight (*p* < 0.05).

**Table 2:** Effect of *Bifidobacterium animalis* subsp. *lactis* XLTG11 and 5-ASA on the body weight of mice.

**Groups**	**Starting weight**	**Final weight**
NC	20.11 ± 0.4	25.47 ± 0.2
MC	20.04 ± 0.1	22.07 ± 0.2**
XLTG11	20.13 ± 0.3	23.11 ± 0.2#
5-ASA	20.19 ± 0.4	23.99 ± 0.3#
XLTG11+5-ASA	20.10 ± 0.3	24.53 ± 0.2##

NC indicates normal control group. MC indicates model control group. XLTG11, 5-ASA, XLTG11+5-ASA indicate the groups in which mice were gavaged with a certain amount of *Bifidobacterium animalis* subsp. *lacti* XLTG11, 5-ASA, and *Bifidobacterium animalis* subsp. *lacti* XLTG11 in combination with 5-ASA, respectively. *p < 0.05 and **p < 0.01 indicate different statistical significances compared with NC group; #p < 0.05 and ##p < 0.01 indicate different statistical significances compared with MC group (*n* = 12).

**Figure 1:**
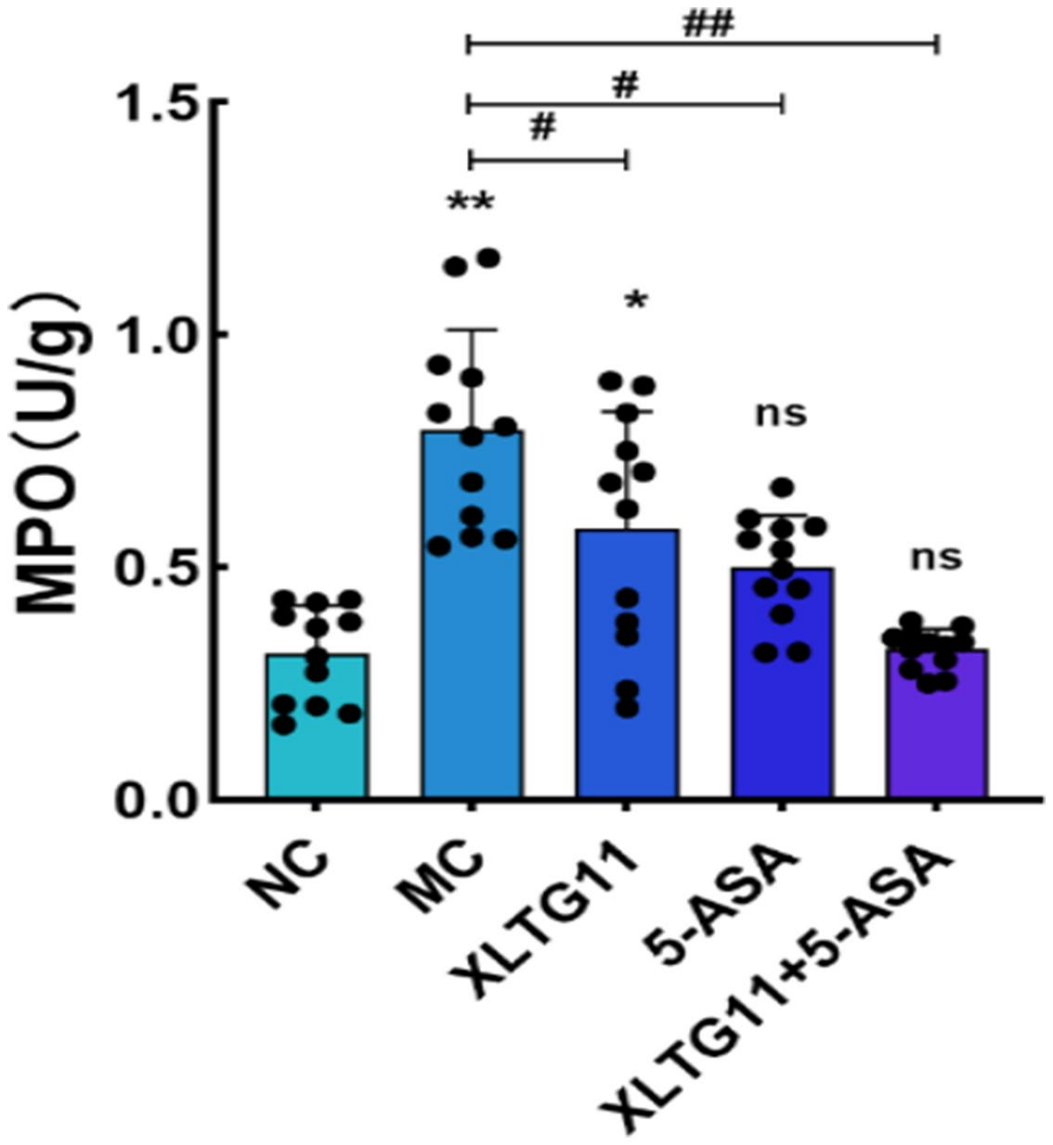
Effect of *Bifidobacterium animalis* subsp. *lactis* XLTG11 and 5-ASA on MPO viability in mice. MPO indicates myeloperoxidase activity. NC indicates normal control group. MC indicates model control group. XLTG11, 5-ASA, XLTG11+5-ASA indicate the groups in which mice were gavaged with a certain amount of *Bifidobacterium animalis* subsp. *lacti* XLTG11, 5-ASA, and *Bifidobacterium animalis* subsp. *lacti* XLTG11 in combination with 5-ASA, respectively. ns, *p < 0.05, and **p < 0.01 indicate different statistical significances compared with NC group; #*p* < 0.05and ##*p* < 0.01 indicate different statistical significances compared with MC group (*n* = 12).

Myeloperoxidase (MPO) is a pro-inflammatory enzyme secreted by neutrophils during inflammation and serves as a marker of gut inflammation. The MPO activity was measured, and the results are presented in Figure 1. As shown in the figure, MPO activity in the MC group was significantly higher compared to the NC group (*p* < 0.01), confirming the successful establishment of the ulcerative colitis model. Treatment with XLTG11 and 5-ASA significantly reduced MPO activity compared to the MC group (*p* < 0.05). Furthermore, the combination treatment of XLTG11+5-ASA led to a more pronounced decrease in MPO activity (*p* < 0.01).

### 3.2 Effect of different groups on histopathology in mice

**Figure 2:**
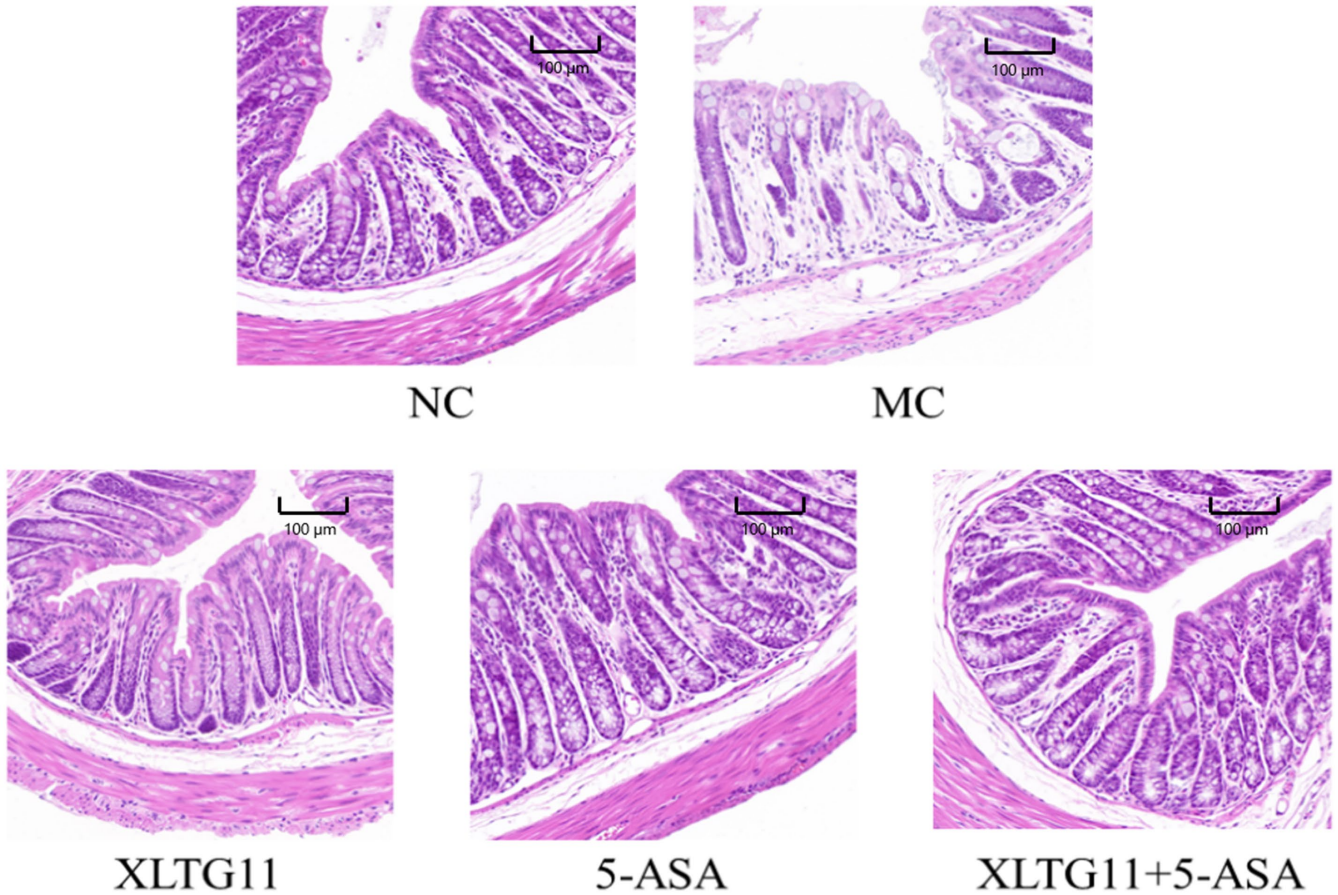
Effect of *Bifidobacterium animalis* subsp. *lactis* XLTG11 and 5-ASA on pathological changes in the mouse colon. NC indicates normal control group. MC indicates model control group. XLTG11, 5-ASA, XLTG11+5-ASA indicate the groups in which mice were gavaged with a certain amount of *Bifidobacterium animalis* subsp. *lacti* XLTG11, 5-ASA, and *Bifidobacterium animalis* subsp. *lacti* XLTG11 in combination with 5-ASA, respectively (*n* = 12). Scale bar = 100 μm.

HE staining results were used to visualize the histopathology of the colon in mice. As shown in Figure 2, the NC group exhibited abundant goblet cells, well-organized intestinal glands, and uniform submucosal interstitial space without inflammatory cell infiltration. In contrast, the MC group showed severe edema, extensive necrosis of the mucosal layer, loss of crypt structure, and diffuse infiltration of inflammatory cells. Compared to the MC group, treatment with XLTG11, 5-ASA, and the combination of both significantly reduced inflammatory cell infiltration. Additionally, these treatments alleviated edema, preserved mucosal crypt structures, and improved the histopathological changes induced by DSS.

### 3.3 Intestinal permeability

**Figure 3:**
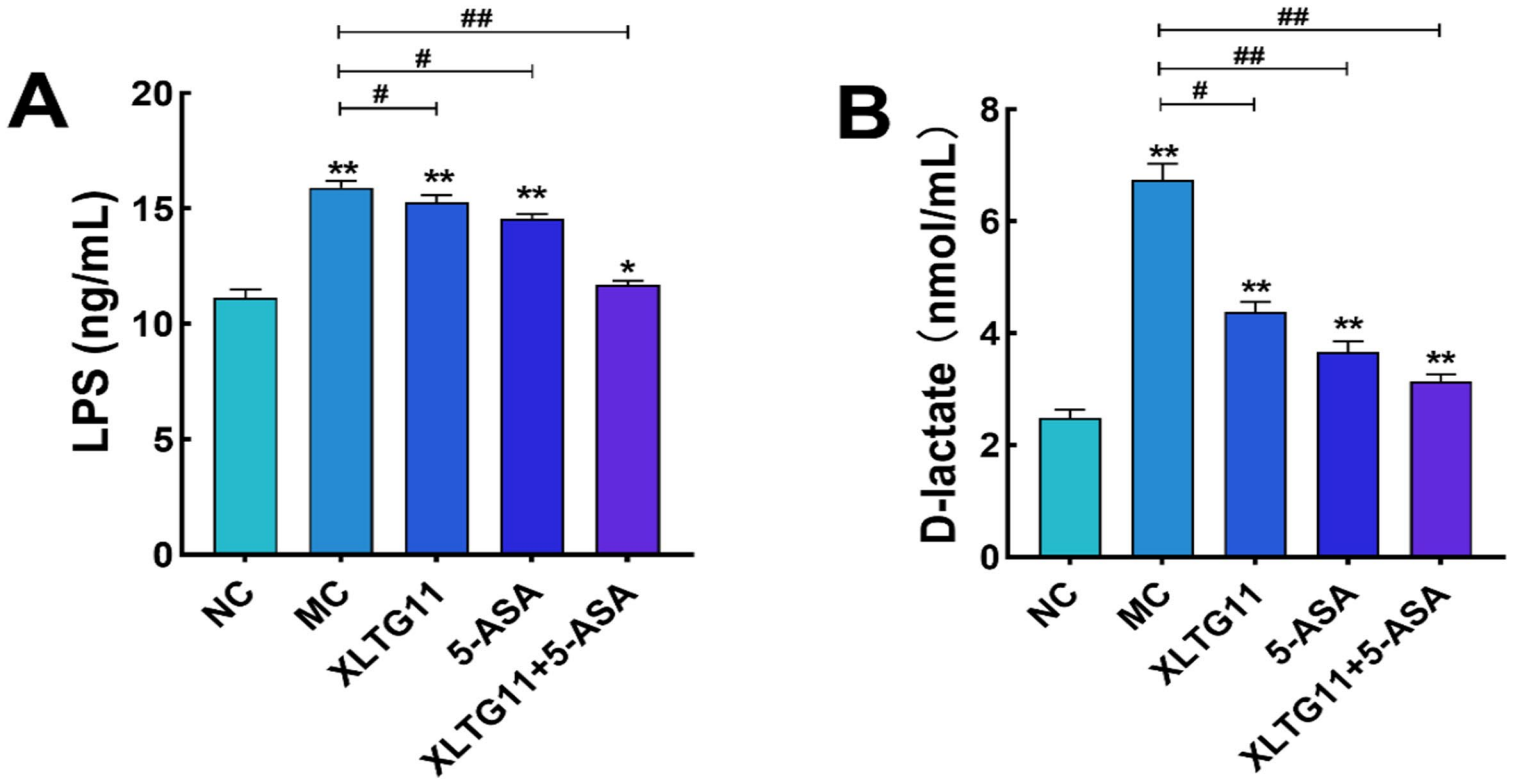
Effects of *Bifidobacterium animalis* subsp. *lactis* XLTG11 and 5-ASA on the serum levels of lipopolysaccharide **(A)** and D-lactic acid in model mice **(B)**. NC indicates normal control group. MC indicates model control group. XLTG11, 5-ASA, XLTG11+5-ASA indicate the groups in which mice were gavaged with a certain amount of *Bifidobacterium animalis* subsp. *lacti* XLTG11, 5-ASA, and *Bifidobacterium animalis* subsp. *lacti* XLTG11 in combination with 5-ASA, respectively. ns, *p < 0.05, and **p < 0.01 indicate different statistical significances compared with NC group; #*p* < 0.05 and ##*p* < 0.01 indicate different statistical significances compared with MC group (*n* = 12).

In this study, ELISA kits were used to measure the levels of LPS and D-lactic acid in the serum of mice, which helped evaluate the effects of different treatments on intestinal barrier permeability. As shown in Figure 3A, the serum LPS level was significantly higher (*p* < 0.01) in the MC group compared to the NC group. After treatment with XLTG11 or 5-ASA, the LPS levels were significantly decreased (*p* < 0.05) compared to the MC group, with the most significant reduction observed in the combination treatment group (XLTG11+5-ASA). Figure 3B shows that the D-lactate content was significantly higher in the MC group than in the NC group (*p* < 0.01). The D-lactate levels were significantly reduced in the XLTG11-treated group (*p* < 0.05), and even more so in the 5-ASA and XLTG11+5-ASA combination-treated groups (*p* < 0.01) compared to the MC group. These results indicate that the combination treatment of XLTG11+5-ASA effectively reduced intestinal barrier permeability, and its therapeutic effect was significantly superior to that of either treatment alone.

### 3.4 Measurement of the intestinal barrier

**Figure 4:**
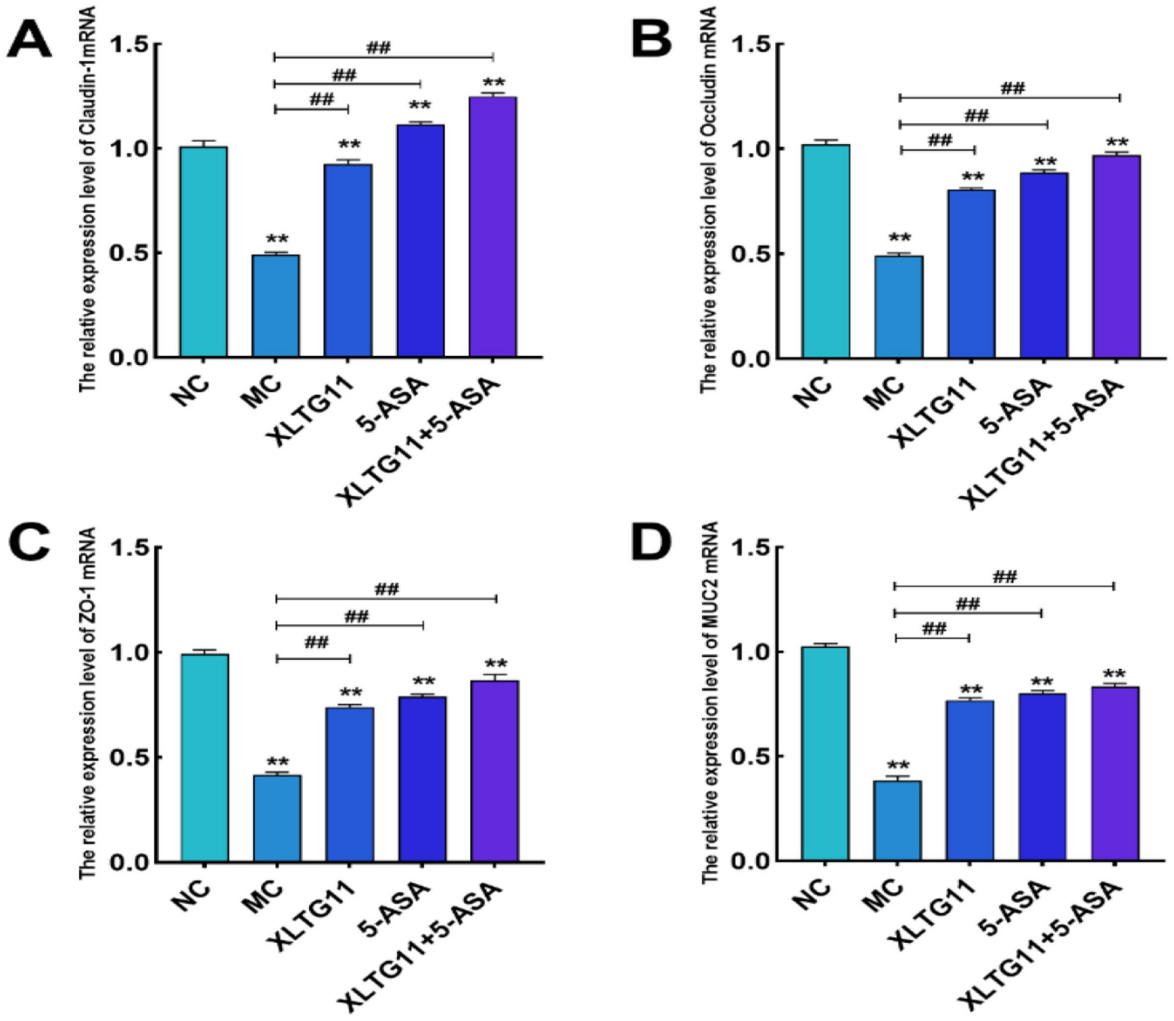
Effects of *Bifidobacterium animalis* subsp. *lactis* XLTG11 and 5-ASA on the expression levels of intestinal barrier-related genes in mice. **(A)** Claudin-1; **(B)** Occludin; **(C)** ZO-1; **(D)** MUC2. NC indicates normal control group. MC indicates model control group. XLTG11, 5-ASA, XLTG11+5-ASA indicate the groups in which mice were gavaged with a certain amount of *Bifidobacterium animalis* subsp. *lacti* XLTG11, 5-ASA, and *Bifidobacterium animalis* subsp. *lacti* XLTG11 in combination with 5-ASA, respectively. ns, *p < 0.05, and **p < 0.01 indicate different statistical significances compared with NC group; #*p* < 0.05 and ##*p* < 0.01 indicate different statistical significances compared with MC group (*n* = 12).

To evaluate the effects of XLTG11 and 5-ASA on intestinal barrier function in mice after DSS treatment, we measured the relative mRNA expression levels of colonic tight junction proteins and mucins, as shown in Figure 4. The relative mRNA expression levels of Claudin-1, Occludin, ZO-1, and MUC2 were significantly lower in the MC group compared to the NC group (*p* < 0.01), indicating impairment of the colonic epithelial integrity. In comparison to the MC group, the mRNA expression levels of Claudin-1, Occludin, ZO-1, and MUC2 were significantly higher in all treatment groups (*p* < 0.01). Notably, the combination treatment of XLTG11+5-ASA showed the most significant improvement in intestinal barrier repair.

### 3.5 Measurement of cytokines

**Figure 5:**
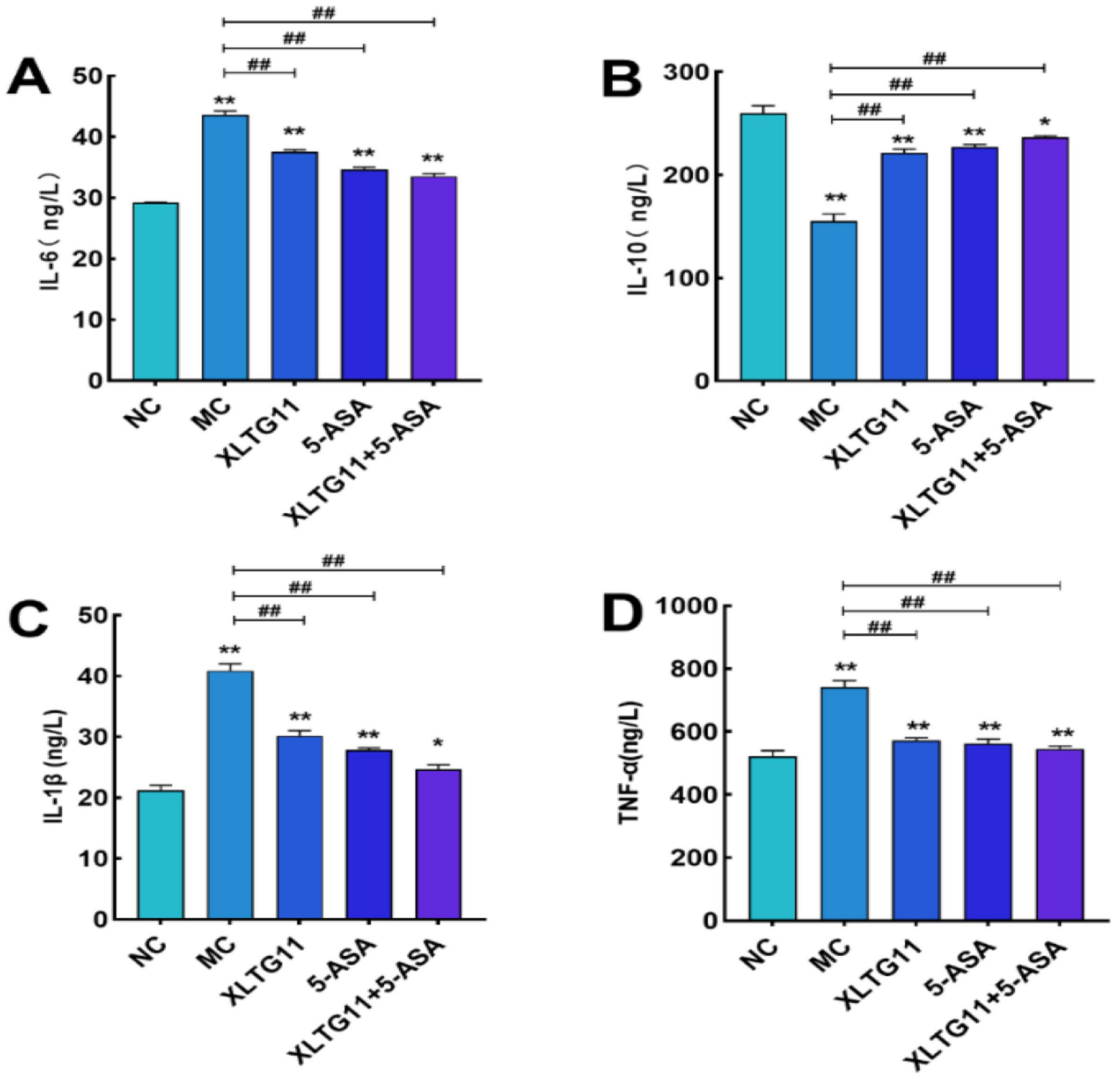
Effect of *Bifidobacterium animalis* subsp. *lactis* XLTG11 and 5-ASA on the level of inflammatory factors in mice. NC indicates normal control group. **(A)** IL-6; **(B)** IL-10; **(C)** IL-1β; **(D)** TNF-α. MC indicates model control group. XLTG11, 5-ASA, XLTG11+5-ASA indicate the groups in which mice were gavaged with a certain amount of *Bifidobacterium animalis* subsp. *lacti* XLTG11, 5-ASA, and *Bifidobacterium animalis* subsp. *lacti* XLTG11 in combination with 5-ASA, respectively. ns,*p < 0.05, and **p < 0.01 indicate different statistical significances compared with NC group; #*p* < 0.05 and ##*p* < 0.01 indicate different statistical significances compared with MC group (*n* = 12).

In this experiment, the levels of TNF-*α*, IL-1β, IL-6, and IL-10 in the mouse colon were measured using ELISA kits to assess the impact of different treatments on inflammatory factors in murine enteritis. As shown in Figure 5, the levels of the pro-inflammatory cytokines TNF-α, IL-1β, and IL-6 were significantly higher in the MC group compared to the NC group (*p* < 0.01), while the anti-inflammatory cytokine IL-10 was significantly lower (*p* < 0.01). Treatment with XLTG11, 5-ASA, or the XLTG11+5-ASA combination significantly reduced the levels of TNF-α, IL-1β, and IL-6 (*p* < 0.01) and increased IL-10 levels, with the most pronounced effect observed in the XLTG11+5-ASA combination group.

### 3.6 Measurement of short-chain fatty acids (SCAFs)

**Figure 6:**
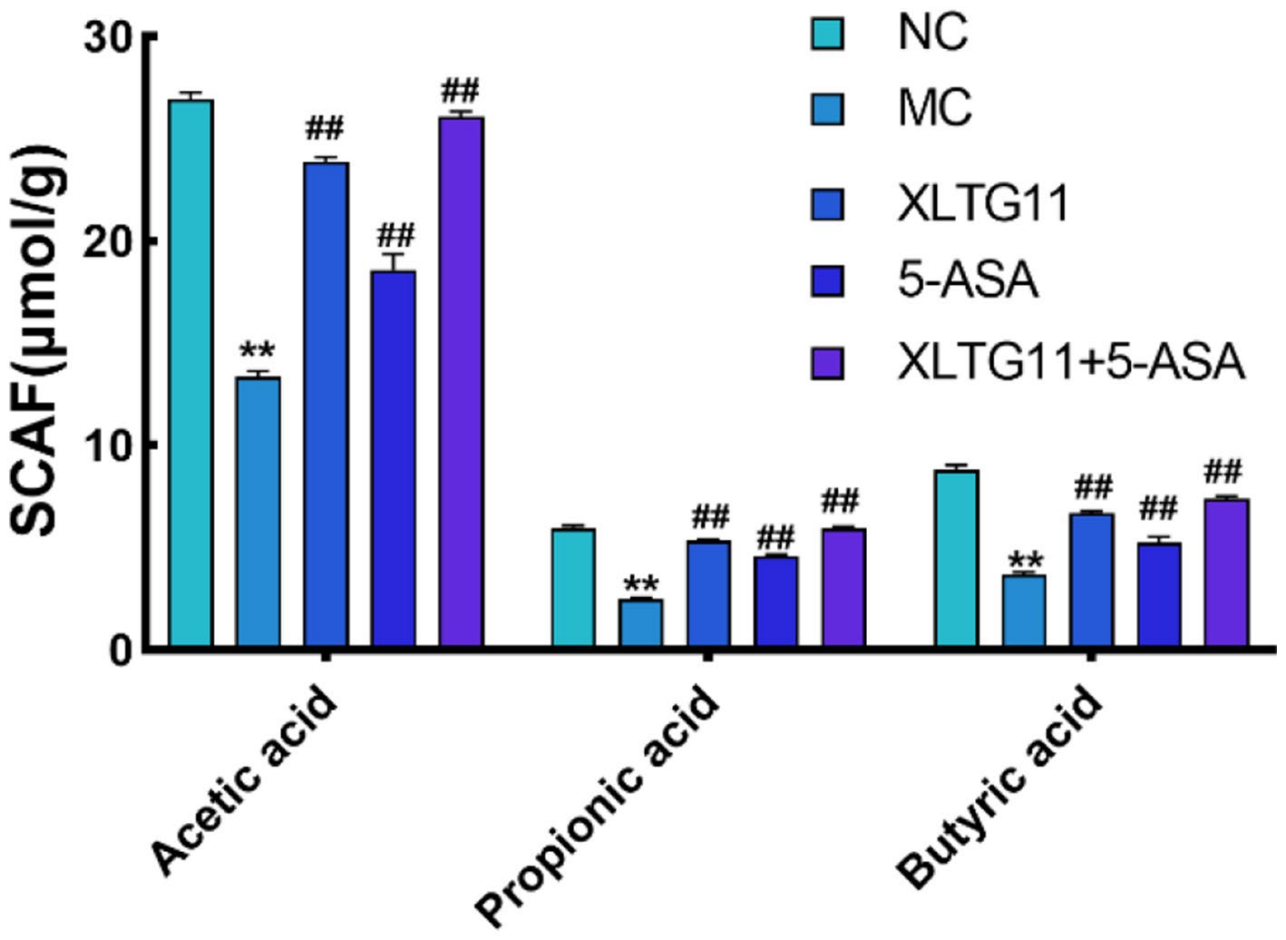
Effect of *Bifidobacterium animalis* subsp. *lactis* XLTG11 and 5-ASA on short-chain fatty acid content in mouse colon. NC indicates normal control group. MC indicates model control group. XLTG11, 5-ASA, XLTG11+5-ASA indicate the groups in which mice were gavaged with a certain amount of *Bifidobacterium animalis* subsp. *lacti* XLTG11, 5-ASA, and *Bifidobacterium animalis* subsp. *lacti* XLTG11 in combination with 5-ASA, respectively. ns, *p < 0.05, and **p < 0.01 indicate different statistical significances compared with NC group; #*p* < 0.05 and ##*p* < 0.01 indicate different statistical significances compared with MC group (*n* = 12).

SCFAs are key metabolites produced by gut microbiota, and changes in their concentrations can reflect the balance of gut microbiota in the host organism (Morrison and Preston, 2016). The changes in SCFA content in the intestine are shown in Figure 6. Compared to the NC group, the levels of acetic acid, propionic acid, and butyric acid were significantly reduced in the MC group (*p* < 0.01). In contrast, all treatment groups, including XLTG11, 5-ASA, and the XLTG11+5-ASA combination, significantly increased the levels of acetic acid (*p* < 0.01), propionic acid, and butyric acid, with the most pronounced effect observed in the combined treatment group.

### 3.7 Effect of different groups on the intestinal microorganisms of mice

**Figure 7:**
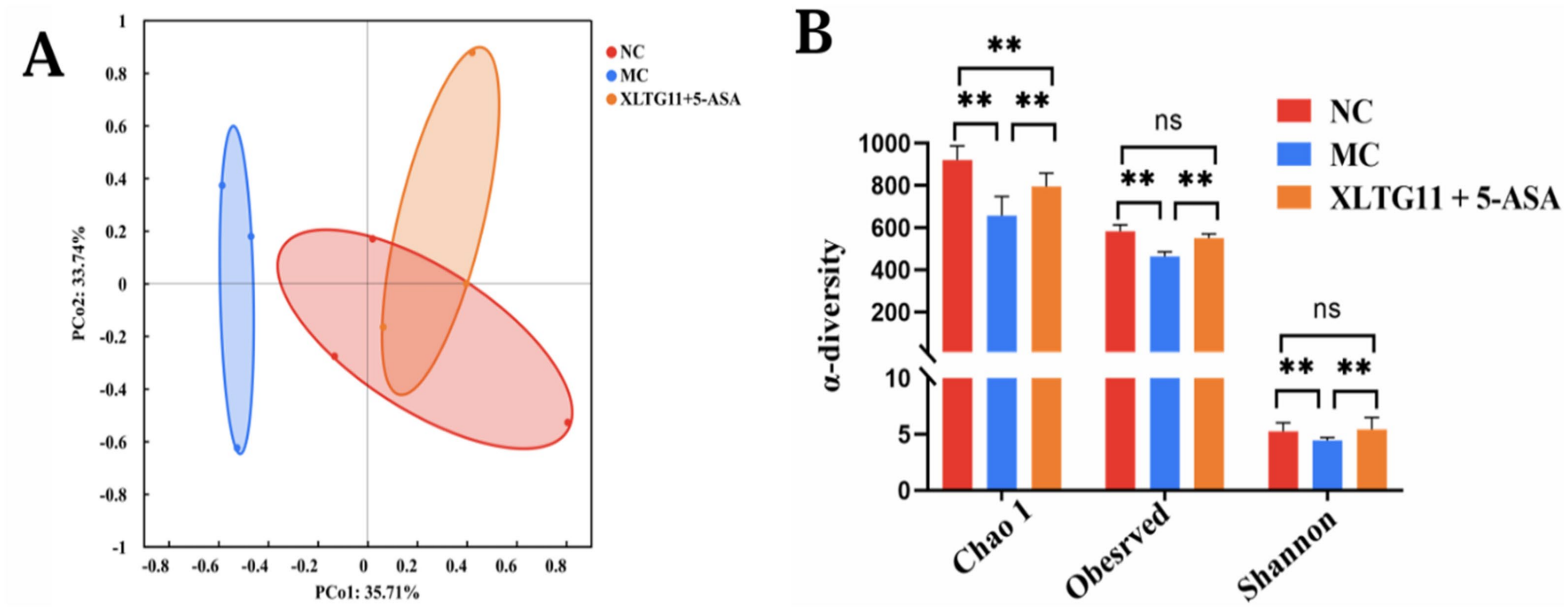
Effect of *Bifidobacterium animalis* subsp. *lactis* XLTG11 and 5-ASA on intestinal microorganisms composition. **(A)** PCoA analysis. **(B)** *α*-diversity of gut microbes. NC indicates normal control group. MC indicates model control group. XLTG11+5-ASA indicate the groups in which mice were gavaged with a certain amount of *Bifidobacterium animalis* subsp. *lacti* XLTG11 in combination with 5-ASA, respectively. ns, *p < 0.05, and **p < 0.01 indicate different statistical significances compared with NC group (*n* = 12).

The results of 16SrRNA sequencing showed the effect of probiotics XLTG11 combined with 5-ASA treatment on intestinal microbial diversity in mice. PCoA analysis showed significant differences between NC, MC and XLTG11+5-ASA groups (Figure 7A). After DSS treatment, the *α* diversity index (including Chao1 index, observation index and Shannon index) of intestinal microorganisms in mice was significantly decreased (Figure 7B). The increase of microαbiodiversity index was observed after XLTG11+5-ASA administration.

**Figure 8:**
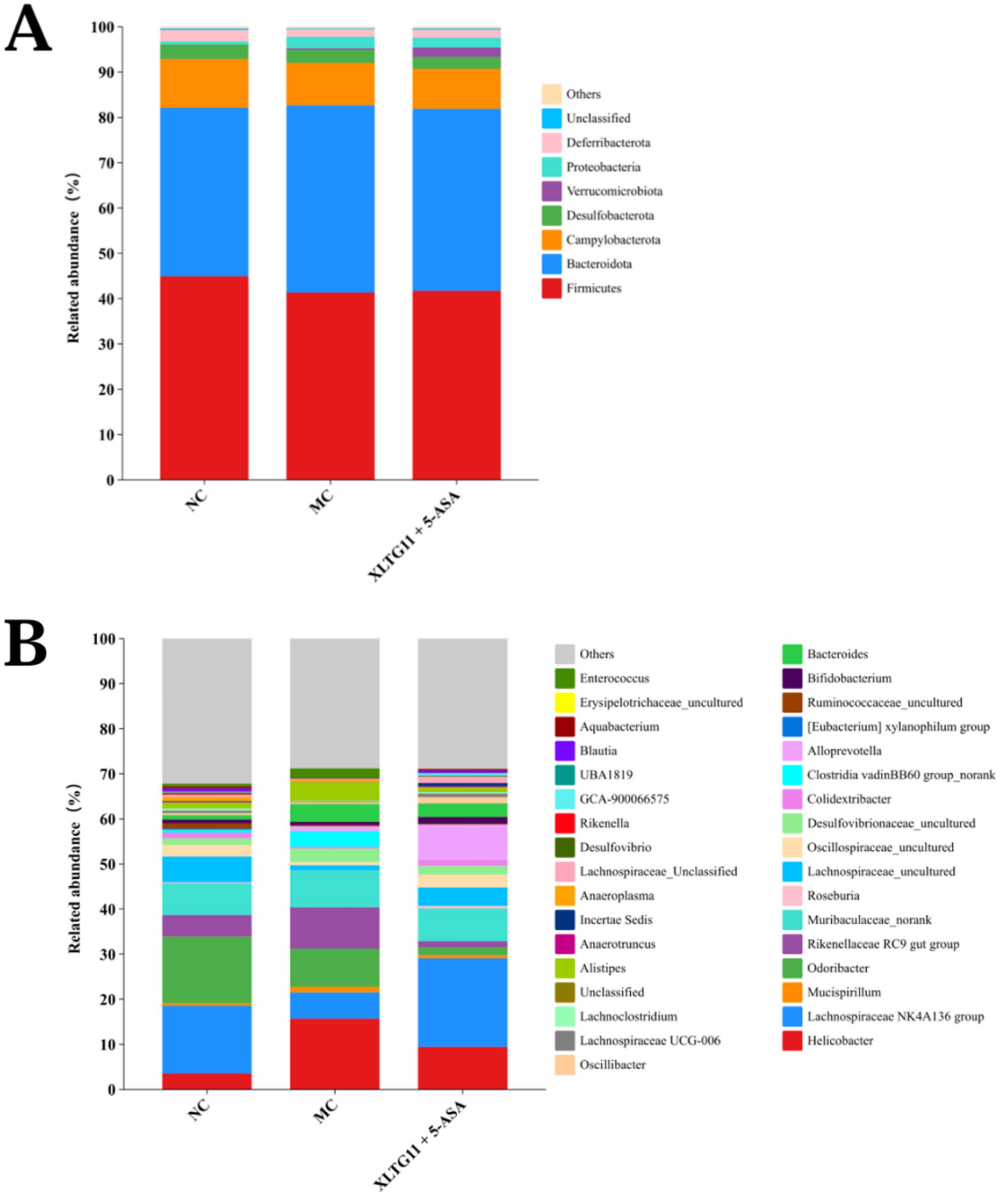
Effect of *Bifidobacterium animalis* subsp. *lactis* XLTG11 and 5-ASA on intestinal microorganisms composition. **(A)** Phylum level. **(B)** Genus level. NC indicates normal control group. MC indicates model control group. XLTG11+5-ASA indicates the group in which mice were gavaged with a certain amount of *Bifidobacterium animalis* subsp. *lacti* XLTG11 in combination with 5-ASA, respectively (*n* = 12).

At the phylum level, Firmicutes and Bacteroidetes were the dominant bacterial groups in the intestinal microbiota of mice across all experimental groups, with no significant differences observed (Figure 8). At the genus level, compared to the NC group, the MC group showed an increase in the relative abundance of *Helicobacter*, *Muribaculaceae_norank*, *Desulfovibrionaceae_uncultured*, *Bacteroides*, and *Alistipes*. Conversely, the relative abundance of *Roseburia* and *Bifidobacterium*, both of which are considered beneficial for IBD, decreased in the MC group. Interestingly, treatment with the XLTG11+5-ASA combination normalized the changes in these genera, restoring their relative abundance to levels similar to those in the NC group. These results suggest that XLTG11+5-ASA combination therapy may help reshape the intestinal microbiota of IBD mice to resemble that of the NC group.

## 4 Discussion

Mesalazine is commonly used to treat inflammatory bowel diseases (IBD), such as ulcerative colitis and Crohn’s disease, rather than stomach ulcers. The drug also inhibits neutrophil lipoxygenase activity, thereby suppressing phagocytosis and migration of oxygen free radicals (Zhang et al., 2019). Additionally, mesalazine acts directly on the affected area to reduce inflammatory factors, alleviate intestinal mucosal inflammation, and promote the healing of gastric ulcers, showing some efficacy in this regard (Beiranvand et al., 2021). However, the drug is prone to recurrence and often results in poor long-term therapeutic outcomes (Le Berre et al., 2020). Therefore, it is typically recommended to combine mesalazine with other therapeutic approaches.

Previous studies have demonstrated that *Bifidobacterium animalis* subsp. *lactis* XLTG11 (XLTG11) has preventive and therapeutic effects on colitis (Wang et al., 2021). This study aimed to investigate the potential of XLTG11 in combination with mesalazine (5-ASA) to ameliorate IBD. Specifically, we evaluated the effects of the XLTG11+5-ASA combination on IBD by measuring changes in the intestinal microbiota, conducting histopathological analyses, assessing intestinal barrier function, and measuring cytokine levels and myeloperoxidase (MPO) activity. MPO activity is a reliable marker for the degree of neutrophil infiltration and reflects the level of intestinal inflammation induced by DSS in mice (Yanez-Lemus et al., 2022). Treatment with XLTG11, 5-ASA, and XLTG11+5-ASA all significantly reduced MPO activity, with the most pronounced effect observed in the XLTG11+5-ASA group. Furthermore, XLTG11+5-ASA also effectively improved the pathological changes caused by DSS, consistent with findings from other studies (S. Li et al., 2024). At the molecular level, lipopolysaccharide (LPS) and D-lactic acid are sensitive indicators of intestinal barrier damage (Song et al., 2009). The combination treatment significantly reduced the levels of LPS and D-lactic acid, thereby improving intestinal permeability.

Tight junctions, located on the surface of intestinal epithelial cells, play a crucial role in forming a mucosal barrier. Aberrant expression of tight junction proteins disrupts this barrier, serving as an initiator of intestinal inflammation (Schleimer and Berdnikovs, 2017). A deficiency in tight junctions increases intestinal barrier permeability, allowing bacteria and harmful antigens to invade, which in turn triggers intestinal inflammation. Key tight junction proteins include Occludin, Claudins, ZO-1, and ZO-2, all of which are integral components of the intestinal mucosal barrier and regulate the permeability and integrity of the intestinal epithelium (Yang et al., 2015). Claudins are particularly important for maintaining intestinal epithelial homeostasis and regulating inflammation (Barmeyer et al., 2017). Occludin, a transmembrane protein, plays a vital role in maintaining epithelial barrier function and regulating intestinal permeability (Otani and Furuse, 2020; Yu et al., 2010). Mucins, such as MUC1 and MUC2, form an additional protective layer on the surface of intestinal epithelial cells. Disruption in the expression of mucins also contributes to barrier breakdown and inflammation (Johansson et al., 2008). In this study, DSS treatment significantly reduced the relative expression of Claudin-1, Occludin, ZO-1, and MUC2 mRNA compared to the NC group. However, treatment with XLTG11, 5-ASA, and their combination significantly increased the expression of these proteins. Notably, the combination of XLTG11 and 5-ASA showed the most pronounced therapeutic effect, suggesting that this combination can improve intestinal barrier function and alleviate IBD in mice, which is consistent with findings from other studies (Tan et al., 2019).

Inflammatory cytokines are signaling molecules secreted by immune and other cell types that promote inflammation (Siraki, 2021). Key inflammatory cytokines such as TNF-*α*, IL-6, IL-1β, and IL-10 are often used to assess the inflammatory state, with their overexpression commonly associated with inflammation (Jum-ming and Jianxiong, 2007). In the present study, DSS treatment in the MC group resulted in a decrease in IL-10 levels, while TNF-α, IL-1β, and IL-6 levels were significantly elevated in the colonic tissues of mice. TNF-α not only damages intestinal epithelial cells but also induces apoptosis, triggering mucosal injury. Excessive IL-1β further promotes the expression of other inflammatory factors, increases intestinal permeability, and exacerbates mucosal inflammation (Philip et al., 2015). Conversely, IL-10 is an anti-inflammatory cytokine that inhibits the release of pro-inflammatory factors and helps mitigate inflammation (Jesudas et al., 2020). Treatment with XLTG11 and 5-ASA together reversed these effects by significantly reducing TNF-α, IL-1β, and IL-6 levels while increasing IL-10, which is consistent with findings from Yan et al. (2019).

Short-chain fatty acids (SCFAs) have been shown to possess anti-inflammatory and anti-tumor effects, and are closely linked to host metabolism (Fernández et al., 2016). As a primary energy source for intestinal epithelial cells, SCFAs promote cell proliferation and differentiation, enhance the intestinal chemical barrier, and regulate immune function in the intestinal epithelium (Li et al., 2018; Makki et al., 2018). In this study, compared to the MC group, acetic acid, propionic acid, and butyric acid levels were significantly higher in the combined XLTG11 and 5-ASA treatment group, suggesting that this combination effectively alleviates inflammatory bowel disease (IBD) by promoting SCFA production.

Intestinal dysbiosis plays a key role in the etiology of IBD (Vemuri et al., 2017; Garcia-Mantrana et al., 2018). IBD typically leads to dysbiosis. In this study, DSS reduced the diversity of intestinal microbiota in mice, leading to dysbiosis. However, XLTG11 combined with 5-ASA treatment increased the diversity of intestinal microbiota in mice and restored the microbial community structure. Additionally, the relative abundance of Helicobacter and Desulfovibrionaceae at the genus level increased in the MC group compared to the NC group. Studies have shown that Helicobacter and Desulfovibrionaceae can secrete lipopolysaccharides, thereby exacerbating ulcerative colitis and Crohn’s disease (Liu and Wang, 2013). The XLTG11+5-ASA group reduced the relative abundance of these pathogens. Additionally, compared to the MC group, the XLTG11+5-ASA group increased the abundance of beneficial microorganisms, such as Roseburia and Bifidobacterium. Roseburia has been shown to prevent intestinal inflammation and maintain energy homeostasis through metabolite production (Nie et al., 2021). Bifidobacterium is known to be an important component of the intestinal microbiota, which can effectively regulate gut microorganisms, promote short-chain fatty acid (SCFA) production, enhance the intestinal barrier, and help alleviate IBD (Yao et al., 2021). In summary, XLTG11 combined with 5-ASA effectively restores DSS-induced intestinal dysbiosis in mice.

## 5 Conclusion

*Bifidobacterium animalis* subsp. *lactis* XLTG11, combined with mesalazine, significantly improved the condition of mice with IBD, reducing colonic tissue damage, decreasing intestinal permeability, and increasing the mRNA expression of tight junction proteins, as well as the content of short-chain fatty acids (SCFAs). Importantly, XLTG11 combined with mesalazine effectively restored DSS-induced intestinal dysbiosis in mice, increasing the abundance of beneficial bacteria, reducing harmful bacteria, and normalizing the gut microbiota composition. Moreover, the combination of XLTG11 with 5-ASA was more effective than either the single bacterium or the single drug treatment in alleviating IBD-related symptoms.

This study highlights the significant potential of probiotic agents in the palliative treatment of intestinal diseases. Future high-quality clinical studies are needed to further investigate the mechanisms underlying the beneficial effects of probiotics in IBD management, providing a foundation for their clinical application.

## Data Availability

The raw data supporting the conclusions of this article will be made available by the authors, without undue reservation.
